# Health Care Charges Associated With Physical Inactivity, Overweight, and Obesity

**Published:** 2005-09-15

**Authors:** Louise H Anderson, Brian C Martinson, A. Lauren Crain, Nicolaas P Pronk, Robin R Whitebird, Patrick J O’Connor, Lawrence J Fine

**Affiliations:** HealthPartners Research Foundation; HealthPartners Research Foundation, Minneapolis, Minn; HealthPartners Research Foundation, Minneapolis, Minn; HealthPartners Research Foundation, Minneapolis, Minn; Dr. Pronk is also affiliated with HealthPartners Center for Health Promotion, Minneapolis, Minn; HealthPartners Research Foundation, Minneapolis, Minn; HealthPartners Research Foundation, Minneapolis, Minn; Division of Epidemiology and Clinical Applications, National Heart, Lung, and Blood Institute, Bethesda, Md

## Abstract

**Introduction:**

Physical inactivity, overweight, and obesity are associated with increased morbidity and mortality. The objective of this study was to estimate the proportion of total health care charges associated with physical inactivity, overweight, and obesity among U.S. populations aged 40 years and older.

**Methods:**

A predictive model of health care charges was developed using data from a cohort of 8000 health plan members aged 40 and older. Model cells were defined by physical activity status, body mass index, age, sex, smoking status, and selected chronic diseases. Total health care charges were estimated by multiplying the percentage of the population in each cell by the predicted charges per cell. Counterfactual estimates were computed by reclassifying all individuals as physically active and of normal weight while leaving other characteristics unchanged. Charges associated with physical inactivity, overweight, and obesity were computed as the difference between current risk profile total charges and counterfactual total charges. National population percentage estimates were derived from the National Health Interview Survey; those estimates were multiplied by the predicted charges per cell from the health plan analysis.

**Results:**

Physical inactivity, overweight, and obesity were associated with 23% (95% confidence interval [CI], 10%–34%) of health plan health care charges and 27% (95% CI, 10%–37%) of national health care charges. Although charges associated with these risk factors were highest for the oldest group (aged 65 years and older) and for individuals with chronic conditions, nearly half of aggregate charges were generated from the group aged 40 to 64 years without chronic disease.

**Conclusion:**

Charges associated with physical inactivity, overweight, and obesity constitute a significant portion of total medical expenditures. The results underscore the importance of addressing these risk factors in all segments of the population.

## Introduction

Physical inactivity, overweight, and obesity are strongly associated with increased mortality and morbidity, including cardiovascular disease, diabetes mellitus, degenerative joint disease, depression, and cancer (1-5). The burden of these adverse health risks has become apparent at both the individual and the population levels (6).

The prevalence of overweight, obesity, and physical inactivity has heightened these risk factors as national public health concerns. Recent reports have clearly documented rapidly rising incidence and prevalence of overweight and obesity in the U.S. population during the last four decades (7). The percentage of U.S. adults with a body mass index (BMI) of 30 or higher (i.e., obese) increased from 22.9% (National Health and Nutrition Examination Survey [NHANES] 1988–1994) to 30.5% (NHANES 1999–2000); the percentage with a BMI of 25 or higher (i.e., overweight) increased from 55.9% to 64.5% between the same two study periods (8). Corresponding increases in weight have also been documented in children (9).

The majority of the U.S. population is either completely inactive or does not meet recommended levels of physical activity. Overall physical activity levels for the country measured by the Behavioral Risk Factor Surveillance System (BRFSS) in 2000 indicate that 27.6% of the adult population is completely inactive and 46.2% is insufficiently active (10). Only 26.2% meet the recommended guidelines of 30 minutes of moderate activity at least 5 times per week or at least 20 minutes of vigorous activity 3 times per week (3). Physical activity behavior has remained relatively stable over the past decade. In 1990, 24.3% of the population engaged in recommended levels of physical activity; this prevalence increased only slightly to 25.4% in 1998 (11).

Several studies have directly estimated annual health care costs associated with overweight and obesity. Thompson et al reported that overweight individuals had 10% higher costs than normal-weight individuals (although this percentage was found not to be statistically significant); obese individuals had 36% higher costs than normal-weight individuals (12). Similarly, Quesenberry et al found that overweight individuals had 25% higher health care costs than normal-weight individuals and the extremely obese had 44% higher costs than normal-weight individuals (13). Sturm estimated that obesity was associated with 36% higher inpatient and outpatient costs in a national survey of adults aged 18 to 65 (14). Recently, Finkelstein et al used individual-level national survey data and regression techniques to estimate that 9.1% of total U.S. medical expenditures can be attributed to overweight and obesity (15).

Studies have also estimated the health care costs associated with physical inactivity. Using National Medical Expenditure Survey data from 1987, Pratt et al found that physically inactive individuals had 24% higher health care costs than active individuals (16). Pronk et al, using individual-level health plan data, found that each additional day of physical activity reduced median health care costs by 4.7% and that each unit of BMI increased median charges by 1.9% (17).

Previous research has often relied on aggregate modeling of costs, an approach that has significant drawbacks. Few previous studies have analyzed individual-level charges and risk-factor data, and fewer still have had the capacity to adjust estimates for factors such as age, sex, chronic disease, or smoking status. In this study, we apply these more sophisticated methods and examine the joint effects of physical inactivity, overweight, and obesity. The consideration of joint effects of activity and weight are important because these factors are behaviorally and physiologically intertwined; strategies to address either issue must inherently take account of the other.

The objective of this study was to extend previous research by estimating health care charges associated with physical inactivity, overweight, and obesity and reporting associated charges at a health plan and the national population level. For modeling purposes, we describe the populations using six characteristics: physical activity, weight, chronic disease, smoking status, age, and sex. We report charges associated with the risk factors by age, sex, and presence of two major chronic diseases to gain a deeper understanding about patterns of expenditure and variation in the potential return on investment across different subpopulations.

## Methods

### Health plan data

The study was conducted at HealthPartners, a Minnesota health plan with both owned and contracted clinics; the study was approved by the HealthPartners Institutional Review Board. We used two sources of data: 1) data from a survey of HealthPartners members conducted in 1995 and 2) data on survey respondents extracted from administrative claims with dates of service between January 1, 1996, and December 31, 1999.

#### Survey sample and procedures

All health plan members aged 40 and older who were enrolled in the plan on December 15, 1994, were potential subjects (N = 200,145). The sample was comprised of three strata of members: one group had been diagnosed with none of four chronic conditions; a second group had been diagnosed with one of four chronic conditions; and a third group had been diagnosed with two or more of four chronic conditions. The four conditions were selected because of their impact on health care charges and their frequency in the over-40 population: diabetes, heart disease, hypertension, and dyslipidemia. Conditions were assigned to members based on 1994 administrative data using *International Classification of Diseases, Ninth Revision, Clinical Modification (ICD-9-CM)* coding and pharmacotherapy databases. Further information on the survey methods is available elsewhere (17).

From the group of potential subjects, a study population was selected; the study population was composed of a stratified random sample of 8000 individuals aged 40 and older. From the 158,415 members with none of the four chronic conditions, a random sample of 3000 (1.9%) subjects was selected; from the 34,159 members who had one of the four conditions, a random sample of 2500 (7.3%) subjects was selected; and from the 7571 members who had two or more of the four conditions, a random sample of 2500 (33.0%) was selected.

Study subjects were mailed a survey and followed up by telephone using standard survey methodology (18). The survey included questions related to physical activity, height, weight, chronic disease, smoking, age, and sex. Survey items and scales were drawn from standard survey tools such as the BRFSS, which have been shown to be reliable and valid (19). Survey response rate was 79% (5977/7535). Survey respondents were older and more likely to be female than nonrespondents. Formal analysis of respondents and nonrespondents is available elsewhere (17).

#### Health plan administrative claims data

Survey data were linked to administrative health care claim data, including professional and hospital claims. Pharmacy charges were excluded from analysis because a subset of the subjects did not have a pharmacy benefit. Charges were standardized to 1997 U.S. dollars. Completeness of the data was estimated to be high: 95% or more of all care that members received was provided within the health plan (20).

#### Observations included in the analysis

We selected observations with complete survey data on all study variables and at least 1 month of enrollment during the period between January 1, 1996, and December 31, 1999, resulting in 4674 observations for analysis. Decrements from the initial sample of 8000 were as follows: 533 were unable to complete the survey because of death, disenrollment, or language problems; 159 were excluded because of a proxy respondent; 1309 did not respond; 716 were missing data on study variables; 357 were not enrolled in the prediction period; and 252 were excluded because they were nonwhite. The number of respondents who identified themselves as nonwhite was insufficient to include race in our model, so we excluded race as a factor and restricted our analysis to white respondents.

Of the 4674 observations used in the analysis, 4372 (93.5%) had 12 or more months of enrollment during 1996 through 1999. The distribution of annualized charges was similar whether members had short (less than 12 months) or long (greater than 12 months) lengths of follow-up enrollment. Therefore, no adjustment for length of follow-up was made in our analysis.

Our weighted sample had a two-tailed power of 80% (a = .05) to detect a change of 0.004 in r^2^ from the addition of one independent variable, adjusted for a nine-variable model with an r^2^ = 0.10.

#### Dependent variable

The dependent variable was the log of average annualized health care charges for the period 1996 through 1999, including physician, inpatient, and outpatient charges. Average annualized charges for individuals in our health plan sample, weighted for sampling strata, were $4928. We used averaged annualized charges over a 4-year period to avoid a mass of observations with zero charges and to smooth individual year-to-year random variation. The distribution of charges was highly skewed, so we transformed to a log scale. On the log scale, model residuals looked roughly normal.

#### Independent variables

We used categorical variables for physical activity, BMI, chronic disease, smoking status, age, and sex. The variables and categories were defined as follows: for physical activity, the categories were active, low activity, and inactive; for body mass index, the categories were normal, overweight, and obese; for chronic disease, the categories were yes (for diabetes, heart disease, or both) and no; for smoking status, the categories were never, former, and current; for age, the categories were 40 to 49 years, 50 to 64 years, and 65 years and older.

Data were self-reported through the member survey in 1995. Physical activity status was coded based on respondents' report of how many days in the past week they "have gotten a total of 30 minutes or more" of physical activity. We used three levels of physical activity to examine the contrast among inactive members (zero days per week), members with low activity (1 to 3 days per week), and members meeting recommended guidelines (4 or more days per week). BMI was calculated from self-reported height and weight (kg/m^2^). BMI was categorized into three levels: normal (less than 25.0), overweight (greater than or equal to 25.0 but less than 30.0), and obese (greater than or equal to 30.0). Smoking status was assessed using standard BRFSS questions and classified as never, former, or current smoker (19).

Study subjects were classified as having chronic disease if they indicated that they had been told by a health professional that they had heart trouble, diabetes, impaired glucose tolerance, or borderline diabetes. We used these classifications in our model to control for two major chronic diseases: heart disease and diabetes. Our purpose was to estimate an upper bound on the charges associated with the modifiable factors of physical activity behavior and overweight or obesity. We controlled for the presence of major chronic disease at baseline because chronic disease is not generally modifiable.

By categorizing independent variables, we may have lost potentially valuable information. However, a main objective of this study was to apply predicted charges to representative samples of health plan and national populations. Hence, the analysis required using categorical variables in our model that would also describe the populations.

### Analytic model 

Starting with health plan data, we constructed a multivariate linear model using the log of average annualized health care charges as the dependent variable, physical activity status and BMI as predictor variables, and chronic disease, smoking status, age, and sex as covariates. Observations were weighted based on the sampling probability and probability of survey response to obtain population estimates. The regression model was based on previously published work (17). We used model coefficients to compute predicted health care charges for 324 cells as defined by categories of physical activity, BMI, and the covariates.

We retransformed the predicted value from the log scale to the dollar scale using the Duan smearing estimator method with multiple smearing factors by sex, chronic disease, and smoking status (21). A single smearing factor is appropriate for retransforming to the dollar scale if the log scale residuals are homoscedastic. If the residuals are heteroscedastic (i.e., they depend on the predictors or covariates), the application of a single Duan smearing factor may give biased expected values (22,23). After reviewing log scale residuals for heteroscedasticity, we confirmed the need for multiple smearing factors.

We tested for interactions among all variables. To ensure accurate estimation of the parameters of interest, we included interactions that were significant at *P* < .10. In particular, we tested for but did not find interaction between the two main effects, physical activity status and BMI. We attributed this result to our study being under-powered to detect an interaction. Our final model included the independent effects of physical activity and BMI.

A single indicator variable for presence of self-reported chronic disease (diabetes or heart disease) was included in the model. This was deemed appropriate because a model with two indicator variables (one for diabetes and one for heart disease) was tested, and results indicated that coefficients for physical inactivity and BMI were similar.

Bootstrapping techniques were used to estimate a 95% confidence interval (CI) for the percentage of charges associated with physical inactivity and overweight or obesity. For each bootstrap iteration, we sampled 4674 observations by strata and with replacement from our analysis observations. We estimated our model on the bootstrap sample and computed the percentage of charges associated with physical inactivity and overweight or obesity. A total of 1000 bootstrap iterations were computed to ensure a stable 95% CI.

### Health plan population 

One goal of this analysis was to estimate the proportion of health care charges associated with physical inactivity, overweight, and obesity for a health plan population. For purposes of simplicity, we assumed a population of 200,000 white members aged 40 and older. We computed the current risk predicted charges for the population by multiplying the model predicted charges for each cell by the percentage of the population in each cell and summing all cells. Hence, this population aggregate reflected the current profile of health behaviors. Next, we computed a counterfactual for the population in a similar manner, but we assigned all members to the lowest risk behaviors: physically active and normal weight, covariates unchanged. Hence, the counterfactual aggregate reflected the lowest risk profile of health behaviors. Finally, the charges associated with physical inactivity, overweight, and obesity were computed as the difference between the current risk aggregate predicted charges and the counterfactual aggregate predicted charges. The associated proportion was computed as the ratio of associated charges to the current risk aggregate predicted charges.

### National population

We extended our analysis to the national level by applying our model predicted charges to a national population. Data from the 2001 National Health Interview Survey (NHIS) were used to represent the U.S. population. The NHIS is an annual, nationally representative telephone survey administered by the National Center for Health Statistics of the Centers for Disease Control and Prevention (CDC). The NHIS samples the civilian, noninstitutionalized household population of the United States. From each family in the NHIS, one adult (aged 18 years or older) was randomly selected. These 33,326 individuals comprised the Sample Adult Core of the 2001 NHIS.

Respondents answered questions about their current levels of cigarette smoking and physical activity. They also reported their height and weight, from which BMI was calculated. Individuals who had been told by a doctor or health professional that they had heart disease or diabetes were considered to have a chronic illness.

In the current study, data limited to whites aged 40 and older were used to estimate the percentage of the U.S. population for each cell defined by the combination of physical activity, BMI, chronic disease, smoking status, age, and sex. The data were weighted to account for the multistage complex sample design of the NHIS. Sample weights also reflected nonresponses, and poststratification adjustments were made for age, sex, and race and ethnicity. The Survey Data Analysis (SUDAAN, Research Triangle Institute, Research Triangle Park, NC) computer program was used to analyze the data.

## Results

### Health plan 

Descriptive statistics of the health plan survey are reported in Tables 1 and 2. Table 1 provides descriptive statistics of physical activity, BMI, and the model covariates. Table 2 provides descriptive statistics for averaged annualized charges. The stratified sampling design allowed for sufficient observations over all combinations of physical activity, BMI, and the covariates to estimate our parameters. A simple random sample of observations would need to be much larger to populate combinations that are naturally sparse.

Model coefficients and associated *t* values are reported in Table 3. The reference category was physically active, normal weight, no chronic disease, never a smoker, male, aged 40 to 49. Main effects of the predictor variables and covariates were as expected. Predicted charges increased with physical inactivity and BMI. Predicted charges were higher for women, for older age categories, and for individuals with chronic disease. Former smokers had higher predicted charges than either nonsmokers or current smokers.

Most interactions among variables were in the expected direction: the higher predicted charges for women decreased with age; predicted charges were higher for members with chronic disease who were former or current smokers; and overweight and obesity had the greatest impact on individuals aged 40 to 49. Interestingly, results indicated no material impact from physical inactivity for women, after adjustment for other factors.

**Health plan charges associated with physical inactivity, overweight, and obesity**

We found that 23.5% of predicted aggregate health care charges were associated with physical inactivity, overweight, and obesity (Table 4). Ninety-five percent of bootstrap iteration results fell between 10% and 34%, indicating our results were significantly different from zero. For a health plan of 200,000 white members aged 40 and older, we estimated a total of $1.12 billion of annualized health care charges in 1997 dollars, of which approximately $263 million was associated with physical inactivity and overweight or obesity (Table 4). The amount and proportion of charges associated with physical inactivity and overweight or obesity were not uniform across health plan members. Table 4 reports results for subpopulations defined by sex, chronic disease, and age. For each subpopulation, Table 4 reports the percentage of the hypothetical health plan population, average annual charges based on the current risk profile, and the proportion of charges associated with physical inactivity and overweight or obesity. The final column of Table 4 reports the total health plan charges associated with physical inactivity and overweight or obesity. Associated charges per year represent health plan expenditures associated with physical inactivity and overweight or obesity for each subgroup, assuming a 200,000-member health plan composed of white members aged 40 and older.

In order of dollar impact, the three subpopulations with the largest associated charges were 1) men aged 50 to 64 with no chronic disease ($44.7 million); 2) men aged 65 and older with chronic disease ($43.7 million) and 3) men aged 40 to 49 with no chronic disease ($41.7 million). These three cells accounted for approximately one half of the hypothetical health plan charges associated with physical inactivity and overweight or obesity. Two of these three large impact groups included nonelderly people that did not self-report either heart disease or diabetes. In fact, nearly half of charges associated with physical inactivity and overweight or obesity were from the groups aged 40 to 64 without chronic disease.

By age group, the subpopulation aged 65 or older had the highest associated charges, reflecting the importance of average charges in the oldest age category. Men had much higher associated charges than women; men accounted for 75% of total associated charges. The associated proportion for members with chronic disease was similar to the associated proportion for members without chronic disease; however, the impact of higher average charges for members with chronic disease resulted in that group generating more than one third of the total charges associated with physical inactivity and overweight or obesity, despite the smaller population (16% of health plan members in this analysis had chronic disease).

### National population

Table 5 shows national and health plan population distributions by physical activity, BMI, chronic disease, smoking status, age, and sex. Nationally, more people were inactive than the health plan population, 39.9% compared with 25.4%. The populations had similar distributions of BMI where 38.5%, 38.6%, and 22.9% of the national population were normal, overweight, and obese, respectively, compared with 39.4%, 39.6%, and 21.0% of health plan members.

The national population had a higher percentage (22.8%) with chronic disease (diabetes, heart disease, or both) than the health plan population (16.4%). Compared with the health plan population, the national population had higher proportions of both current smokers (20.6%, national vs 16.9%, health plan) and people who had never smoked (47.3%, national vs 42.8%, health plan). The national population was almost evenly divided by sex, whereas the health plan population had more women than men.

**National estimate of charges associated with physical inactivity, overweight, and obesity**

Using the same methods as described above for the health plan population, we found that 27% (95% CI, 10%–37%) of national health care charges for individuals aged 40 and older were associated with physical inactivity, overweight, and obesity. The national estimate was higher than the health plan estimate because of the greater percentage of the national population that was less physically active, the greater percentage with chronic disease, and the greater percentage of men.

## Discussion

This study reports significant association between health behaviors and health care charges, generally consistent with previous evidence suggesting that physical inactivity, overweight, and obesity account for an important portion of health care expenditures. The results of this study indicate that physical inactivity, overweight, and obesity are associated with 23% (95% CI, 10%–34%) of all health care charges at a moderately sized Minnesota health plan and 27% (95% CI, 10%–37%) of national health care charges.

The distribution of these charges across defined groups of patients is not uniform. Per- person charges associated with physical inactivity and overweight or obesity are disproportionately higher for individuals who are old, male, and have heart disease, diabetes, or both. Yet despite higher per-person charges among these groups, nearly half of associated charges are incurred in the demographic segments of the health plan population with the largest number of members — members aged 40 to 64 with no heart disease or diabetes. This suggests that a broad, population-wide approach to managing physical inactivity and obesity would be better than a strategy that limits interventions to people with the highest health care charges.

One interesting finding is the observation that after adjusting for other factors in the model, physical activity level was not related to charges among women. There are several possible explanations for this finding. First, it may be that the types of health care services that men of this age use are more related to physical activity than the types of services that women use. Second, it is possible that women and men are differentially misclassified by our measure of physical activity. For example, if the types of physical activity that women typically engage in are less likely to be reported in response to our simple query about physical activity, then we would be more likely to misclassify women than men. In either case, the exploration of sex-related differences in the health impact of physical activity should receive more attention.

The finding of higher health care charges among former smokers (compared with current smokers and people who have never smoked) has been reported elsewhere (17) and is most likely explained by the tendency for smokers who develop health problems to be more likely to quit compared to smokers who remain free of health problems (24).

Current discussions of how to control rapidly rising health care expenditures often focus on modifying payment mechanisms, increasing patient cost-sharing, or carefully managing access to certain types of care. Our data emphasize the potential importance of primary prevention and effective management of overweight and physical inactivity as a cost-control strategy. Effective interventions to ameliorate these problems are available and can be delivered at relatively low cost across a wide range of settings, from physicians' offices to telephone-based case management to community health education using a variety of communication methods (25,26). Despite convincing evidence that adverse health risks increase charges and that improving risk factors lowers subsequent charges, health plans, employers, and other payers have been slow to invest resources in initiatives.

Improvements in weight and physical activity may reduce health care expenditures within 2 to 3 years in some groups of patients (17). Yet payers are reluctant to assume new costs in an age of accelerating medical care cost inflation, and leaders of care-delivery systems may believe that their expertise is more related to high-technology procedural care than to interventions that target lifestyle or behavior (27). The high proportion of overall charges associated with inactivity and overweight may prompt both payers and delivery-system leaders to rethink priorities.

A number of factors limit the interpretation of these data. First, the study was conducted at a single health plan, and our analysis is limited to a white population aged 40 and older, excluding pharmacy charges. Second, the variables considered in our models were derived from survey data and automated administrative databases; they did not include sophisticated physiologic measures. Third, we adjusted our predicted cell-charge estimates for self-reported heart disease or diabetes. Other chronic debilitating diseases might have also been used as adjustors, but we selected heart disease and diabetes because of their prevalence and their established contribution to health care expenditures. Fourth, our national estimates of physical inactivity and BMI reflect 2001 population behaviors.

Fifth, we were unable to report a significant interaction between the two main effects, physical inactivity and BMI. To detect an r^2^ change of 0.01 from an interaction term, with two-tailed power of 80% (α = .05), a sample would need 700 observations per interaction cell. Our weighted sample was too small to achieve that level of power; our sample had two-tailed power of 13% to detect an interaction between physical inactivity and BMI. Therefore, our results do not rule out a significant interaction between physical inactivity and BMI; the question of interaction effects remains open for further study.

Finally, one might argue that physically inactive subjects experience higher health care charges because impairment or health-related problems limit their ability to be active. However, we found that the proportion of charges associated with physical inactivity and overweight or obesity was similar for subjects without self-reported chronic disease compared with subjects reporting chronic disease. Moreover, we tested this hypothesis (data not presented) by estimating a model after excluding subjects whose activities are limited because of impairment or a health-related problem. The model estimated after excluding subjects with limited activity was similar to our model including all subjects; our estimate of health care charges associated with physical inactivity and overweight or obesity did not change. This evidence increases our confidence that reverse causality is not a substantial driver of our study results.

One difficult issue in the interpretation of our study results is the extent to which the associations that we measure are the result of causation. The data allow us to estimate the difference in health care charges between individuals with selected characteristics and individuals without these characteristics. We do not know how changing characteristics will impact future health care expenditures, so we remain uncertain about the extent to which our counterfactual is a good measure of the effect of improved health behaviors on health care charges. Generally, our estimates provide a theoretical upper bound on the amount of resources that may be invested in programs to improve risk-factor profiles.

Our results are interesting and important. We jointly estimate the effects of physical inactivity and overweight or obesity associated with health care charges and find they constitute a significant portion of total health care charges. From a clinical and public health point of view, benefit may be derived by addressing physical inactivity, overweight, and obesity in all segments of the population. However, from a cost-effectiveness point of view, those with behavioral risk factors and the highest health care charges may be the strongest candidates for interventions designed to improve risk profiles and reduce expenditures. More work is needed to explore the potential of behavior interventions as a strategy to improve health and to control accelerating health care charges.

## Figures and Tables

**Table 1:** Demographic Characteristics of Participants in Survey of Health Care Plan Members (N = 4674), Minnesota, 1995

**Variables**	**No. Respondents, Unweighted**	**% Respondents, Weighted^a^**

**Physical activity**		

Active	1613	33.7
Low activity	1759	41.1
Inactive	1302	25.2

**Body mass index, kg/m^2^**		

Normal (<25.0)	1567	39.9
Overweight (25.0-29.9)	1883	39.6
Obese (≥30.0)	1224	20.5

**Chronic condition**		

No	2938	84.9
Yes	1736	15.1

**Smoking status**		

Never	1901	43.0
Former	2100	40.0
Current	673	17.1

**Age, y**		

40-49	1333	43.1
50-64	1713	34.5
≥65	1628	22.5

**Sex**		

Female	2455	55.6
Male	2219	44.4

a Weighted for sampling strata and survey response.

**Table 2:** Average Annualized Charges for Health Plan Members in Dollar and Log Scales, by Body Mass Index and Physical Activity (N = 4674), Minnesota, 1996-1999

	**Average Annualized Charges^a^ $ (SD)**	**Log Average Annualized Charges (SD)**
Overall	4928 (9507)	7.02 (1.25)

**Body mass index, kg/m^2^**		

Normal (<25.0)	3994 (8789)	6.86 (1.33)
Overweight (25.0-29.9)	5239 (10,749)	7.00 (1.30)
Obese (≥30.0)	6146 (8226)	7.38 (1.02)

**Physical activity**		

Active	4240 (7560)	6.96 (1.25)
Low activity	4966 (11,860)	6.91 (1.32)
Inactive	5783 (7953)	7.29 (1.14)

a Weighted for sampling strata and survey response; in 1997 dollars.

**Table 3:** Model^a^ Coefficients and *t* Values, Study on Health Plan Members, Minnesota, 1996-1999

	**Coefficient**	***t* Value**
Intercept	5.4692	29.76
Female	0.9615	5.35
Aged 50-64 y	0.5953	2.70
Aged ≥65 y	1.9174	9.54
Chronic disease present	0.8244	4.47
Former smoker	0.2429	2.69
Current smoker	−0.1603	−1.13
Overweight	0.1978	1.28
Obese	0.7740	5.43
Low activity	0.3292	2.17
Inactive	0.4627	2.70

Female × aged 50-64 y	−0.0510	−0.26
Female × aged ≥65 y	−0.5585	−2.80
Female × with chronic disease	−0.4230	−2.24
Female × low activity	−0.3807	−2.07
Female × inactive	−0.3881	−1.91

Chronic disease present × former smoker	0.1439	0.74
Chronic disease present × current smoker	0.7047	2.60

Overweight × aged 50-64 y	0.0834	0.38
Overweight × aged ≥65 y	−0.2330	−1.09
Obese × aged 50-64 y	−0.5642	−2.47
Obese × aged ≥65 y	−0.4592	−2.03
Model r^2^ = 0.145		

a The reference category is physically active, normal weight, no chronic disease, never a smoker, male, aged 40 to 49.

**Table 4:** Charges Associated with Physical Inactivity (PI), Overweight, and Obesity for Hypothetical Health Plan With 200,000 Members Aged 40 Years and Older and White

**Subpopulation**			**% Health Plan Population**	**Predicted Charges per Member per Year^a^** **($)**	**% Charges Associated With PI, Overweight, and Obesity**	**Hypothetical Plan Charges Associated with PI, Overweight, and Obesity^b^ ($1000s)**

**Sex**	**Chronic Disease**	**Age, y**				
Female	No	40-49	21.8	2100	21.1	19,302
		50-64	16.6	3435	14.3	16,309
		≥65	9.8	6677	5.8	7561
	Yes	40-49	1.5	4888	35.4	5210
		50-64	2.2	6535	18.4	5434
		≥65	3.7	14,025	12.0	12,296
Male	No	40-49	16.5	2963	42.7	41,753
		50-64	12.7	4780	36.9	44,714
		≥65	6.2	13,677	21.3	36,440
	Yes	40-49	2.1	5268	48.9	10,750
		50-64	3.2	8428	37.2	20,136
		≥65	3.7	23,123	25.3	43,736
Overall			100.0	5610	23.5	263,648

a Charges are based on current risk profile.

b 200,000 × column 1 × column 2 × column 3.

**Table 5:** Distribution of Physical Activity, Body Mass Index, and Model Covariates Among Health Plan Population and National Population

**Variables and Categories**	**Population**	

	**% Health Plan Members**	**% National Population**

**Physical activity**		

Active	33.7	29.2
Low activity	40.9	30.8
Inactive	25.4	39.9

**Body mass index, kg/m^2^**		

Normal (<25.0)	39.4	38.5
Overweight (25.0-29.9)	39.6	38.6
Obese (≥30.0)	21.0	22.9

**Chronic disease^a^**		

No	83.6	77.2
Yes	16.4	22.8

**Smoking status**		

Never	42.8	47.3
Former	40.3	32.1
Current	16.9	20.6

**Age, y**		

40-49	41.9	35.8
50-64	34.7	35.7
≥65	23.4	28.5

**Sex**		

Female	55.5	51.2
Male	44.5	48.8

a Diabetes, heart disease, or both.
